# Addressing adolescents’ risk and protective factors related to risky behaviours: Findings from a school-based peer-education evaluation in the Western Cape

**DOI:** 10.1080/17290376.2016.1241188

**Published:** 2016-11-28

**Authors:** Furzana Timol, Mohammed Yacoob Vawda, Arvin Bhana, Benita Moolman, Mokhantso Makoae, Sharlene Swartz

**Affiliations:** ^a^ MA, is a Researcher in the Human and Social Development Unit, Human Sciences Research Council, Pretoria, South Africa; ^b^ MA, is a Lecturer in the School of Built Environment and Development Studies, University of KwaZulu-Natal, Durban, South Africa; ^c^ PhD, is a Chief Specialist Scientist in the Health Systems Research Unit, South African Medical Research Council, Durban, South Africa; ^d^ PhD, is an Honorary Associate Professor in the School of Applied Human Sciences, University of KwaZulu-Natal, Durban, South Africa; ^e^ PhD, is a Senior Research Specialist in the Human and Social Development Unit, Human Sciences Research Council, Pretoria, South Africa; ^f^ PhD, is an African Research Fellow in the Human and Social Development Unit, Human Sciences Research Council, Pretoria, South Africa; ^g^ PhD, is a Research Director in the Human and Social Development Unit, Human Sciences Research Council, Pretoria, South Africa; ^h^ PhD, is an Adjunct Associate Professor in the Department of Sociology, University of Cape Town, Capetown, South Africa

**Keywords:** high school, evaluation, high-risk behaviour, HIV prevention, peer education, South Africa, Lycée, évaluation, comportement à haut risque, prévention du VIH, éducation par les pairs, Afrique du Sud

## Abstract

**Background**: Peer-education programmes aim to bring about attitudinal and behavioural changes in their target audience. In the South African educational context, peer education is a favoured approach in dealing with issues such as HIV and AIDS, sexual decision-making and substance misuse. Given the reliance on peer-education programmes in the educational system, it is important to establish how well they are working. This study aims to assess the effect of an extensive, structured, time-limited, curriculum-based, peer-led educational programme on first-year high school learners in public schools in the Western Cape Province of South Africa. **Method:** The curriculum called ‘Listen Up’ addresses issues such as supporting peers, sexual decision-making, healthy relationships, HIV risk, alcohol misuse and unwanted pregnancy in seven structured sessions. The programme targeted adolescents in Grade 8 growing up in what are considered to be risky environments in public schools in the Western Cape during 2012 and 2013. The intervention was evaluated based on 10 scales sourced from published literature related to the outcome indicators of future orientation, sensation-seeking, self-efficacy in sexual relations, HIV transmission knowledge, HIV prevention knowledge, HIV attitudes, sexual attitudes, decision-making, healthy relationships and social support. Descriptive statistics were used to analyse demographic and community characteristics and analyses of variance were used to detect differences between groups. The surveys were administered to a total of 7709 learners across three waves of the study in 27 peer intervention schools and eight control schools. **Results:** Immediately post intervention, statistically significant differences were noted for the intervention schools when compared to their baseline levels on measures of future orientation, self-efficacy in sexual relations, knowledge regarding HIV transmission, knowledge regarding HIV prevention and knowledge in terms of healthy relationships. Comparing baseline values with results collected between five and seven months post intervention, statistically significant results were noted for self-efficacy in sexual relations and knowledge regarding HIV transmission. **Conclusion:** The findings of this study suggest that peer-education can improve adolescents’ self-efficacy in sexual relations as well as knowledge regarding the transmission of HIV and therefore can contribute to the prevention of HIV transmission among adolescents.

## Résumé

**Aperçu:** Les programmes de l’éducation par les pairs visent à provoquer des changements comportementaux et d’attitudes chez le groupe cible. Dans un contexte d’éducation en Afrique du Sud, l’éducation par les pairs est une approche favorisée dans le fait de faire face aux questions telles que le VIH et SIDA, la prise de décision sexuelle et l’abus des substances. Étant donné la confiance dans les programmes d’éducation par les pairs inscrit dans un contexte du système d’éducation, il est important d’établir leur bonne marche. Cette étude vise à évaluer les effets d’un programme d’éducation extensif, stucturé, à durée limitée, axé sur un programme d’enseignement, mené par les pairs sur les élèves de classe de 6^ème^ (première classe de lycée) des établissements scolaires publics dans la Province du Western Cape en Afrique du Sud. **Méthode**: le Programme d’enseignement appelé ‘Listen Up’ traite des questions telles que l’appui par les pairs, prise de décision sexuelle, de relations saines, le risque du VIH, l’abus d’alcool et les grossesses indésirées dans les sept sessions structurées. Le Programme à pour cible des adolescents en classe de 4^ème^ (troisième classe du secondaire) qui vivent et grandissent dans des quartiers qualifiés d’environnements dangereux dans les établissements scolaires publics du Western cape entre 2012 et 2013. Des statistiques détaillées ont été utilisées pour faire l’analyse des caractéristiques démographiques et des communautés, les analyses de variance ont été utilisées pour déterminer les différences entre les groupes. L’intervention a été évaluée sur la base de dix échelles obtenues des documentations publiées relatives aux indicateurs de résultats sur les orientations futures, la recherche de sensation, l’auto efficacité dans les rapports sexuels, la connaissance de transmission de VIH, la connaissance de prévention de VIH, les attitudes de VIH, les attitudes sexuelles, les prises de décisions, les relations saines, et l’aide sociale. L’enquête a été conduite sur un total de 7.709 élèves à travers trois vagues d’études sur 27 interventions scolaires par les pairs et huit contrôles scolaires. **Résultats**: Les différences post intervention immédiates, statistiquement significatives ont été notées pour les établissements scolaires d’intervention lorsque comparées à leurs niveaux de base de référence sur les mesures de l’orientation future, autoéfficacité dans les rapports sexuels, l’auto efficacité dans les rapports sexuels, la connaissance de transmission de VIH, la connaissance de prévention de VIH et la connaissance en ce qui concerne des relations saines. En comparant les valeurs de base de référence aux résultats collectés entre cinq et sept mois, des résultats post intervention et statistiquement significatifs ont été notés pour , l’autoéfficacité dans les rapports sexuels, la connaissance de transmission de VIH. **Conclusion**: les conclusions de cette étude suggèrent que l’éducation par les pairs peut améliorer l’autoéfficacité dans les rapports sexuels, la connaissance de transmission de VIH chez les adolescents, et peut ainsi contribuer à la prévention de la transmission du VIH chez les adolescents.

## 1. Introduction

Adolescence is a period during which people are likely to engage in behaviours that place them at risk in terms of their health and wellbeing (Romer, 2010; Steinberg, 2008). In South Africa, youths report high levels of risky behaviours, including multiple sex partners, teenage pregnancy, infrequent condom use and substance abuse (Marteleto, Lam, & Ranchhod, 2008; Reddy et al., 2010; Shisana et al., 2014). Engagement in risky behaviours increases the risk of HIV for those engaging in such activities (Shisana et al., 2009). Structural issues such as poverty and high levels of violence and abuse have also been found to increase the likelihood of engaging in risky behaviours contributing to the HIV epidemic (Dinkelman, Lam, & Leibbrandt, 2008; Jewkes, Dunkle, Nduna, & Shai, 2010; Woodson, Hives, & Sanders-Phillips, 2010). In the context of under-resourced communities, providing interventions addressing the issues faced by adolescents is challenging given that there are few resources at the level of communities to specifically address these challenges. The high rates of HIV among youths (UNAIDS, 2013) and the potential for long-standing impacts of health promotion interventions at a younger age suggest that the adolescent phase is an ideal period in which to intervene by promoting healthy behaviours (South African Department of Health, 2012; World Health Organization [WHO], 2005).

The decision to engage in risky behaviours is influenced by a number of risk and protective factors. Protective factors are individual attributes and conditions that have been found to be correlated with a decrease in risky behaviours. For instance, higher HIV and AIDS knowledge has been found to be protective against having unsafe sex (Thanavanh, Harun-Or-Rashid, Kasuya, & Sakamoto, 2013; Voisin, Hotton, Tan, & Diclemente, 2013). Risk factors are those attributes found to be correlated with increased risky behaviours, including higher levels of sensation-seeking, which has been identified as associated with unsafe sexual practices (Hendershot, Stoner, George, & Norris, 2007; Nguyen et al., 2012; Voisin et al., 2013).

Adolescence is usually characterised by an increase in time spent with peers and a greater desire to conform to peer norms, hence it is a time of greater peer influence (Steinberg, 2008). Peer influence is often regarded as synonymous with peer pressure or increased risk (Berten, 2008). However, peer influences can also be considered a protective factor. Through reinforcing positive norms and values, peer influences can contribute towards the healthy development of children and adolescents (Werner, 2000). Peer education has been identified as a health promotion strategy and intervention modality (Shiner, 1999) for addressing risky behaviours. It is therefore an ideal tool for bringing about behavioural changes in adolescents. Peer education is the process whereby trained facilitators assist a subset of the target population to ‘educate their peers in a structured manner, … role-model healthy behaviour, recognise individuals in need of additional help and refer them for assistance, and advocate for resources and services for themselves and their peers’ (Deutsch & Swartz, 2002, p. 244). Peer education is based on the rationale that peers have a stronger influence on individual behaviour due to the level of familiarity and trust and the comfort they are able to provide towards their peers (Campbell & MacPhail, 2002). Using individuals from the target group to act as an ‘agent of change’ to change social norms (Chandan et al., 2008, p. 12), peer education is able to complement other more individualised modes of health promotion and delivery (Visser, 2011).

Peer education has been used in a number of contexts to bring about behavioural changes with the ultimate goal of preventing the spread of HIV (Hope, 2003; Medley, Kennedy, O’Reilly, & Sweat, 2009; Sloan & Myers, 2005). A number of peer-education programmes targeting youths have been implemented in South Africa. While substantial resources have been directed towards these programmes (Deutsch & Swartz, 2002), limited evaluation of peer-education programmes in South African schools has been conducted. Findings from South African studies note peer education to be effective in increasing condom usage and postponing sexual debut (Mash & Mash, 2012), contributing to delaying sexual debut (Visser, 2007), improving knowledge of HIV and AIDS prevention (Warwick & Aggleton, 2004) and improving HIV knowledge and attitudes (Swartz et al., 2012) among others. Evaluations conducted in other developing countries indicated a moderate level of effectiveness (Medley et al., 2009; Ross, Dick, & Ferguson, 2006). The results of a systematic review of 13 peer-education studies in South Africa show a decrease in sexual activity and the transmission of sexually transmitted diseases in some studies; however, these results are not consistent across all studies (Kim & Free, 2008). A number of peer-education programmes were found to be ineffective in meeting any of their desired objectives (Mason-Jones, Mathews, & Flisher, 2011; Mukoma et al., 2009; Sloan & Myers, 2005; Williams et al., 2003). The lack of effects is attributable largely to poor implementation or research design.

## 2. Context and setting of the study

In the Western Cape, as in many other places in South Africa, the social and health effects of racial segregation and economic inequality continue to influence contemporary social life. Peer education has the potential to improve the quality of life for young people by challenging and changing these long-standing, harmful behavioural and attitudinal practices in risky environments. Schools provide the ideal location for obtaining information on adolescents and their behaviours (Reddy et al., 2010), and are significant sites for shifting social norms. Goal 25 of the South African Department of Basic Education’s (2011) Action Plan for 2011 to 2014 emphasises using the school as a location to promote children’s access to services including public health services and psycho-social support. Schools are therefore important settings in which to implement innovative peer-education programmes. Many studies of school-based interventions encounter implementation challenges suggesting the need for new ways of delivering such interventions (Harrison, Newell, Imrie, & Hoddinott, 2010). Flisher et al. (2006) report that peer-education programmes aimed at reducing HIV and AIDS prevalence among school-going adolescents are important for a number of reasons. Within the school environment, a large portion of adolescents are sexually active and this is coupled with substantial peer influence relating to sexual activity. Flisher et al. (2006) argue that the schooling environment provides a key link to the larger community structures in the sense that they are often the first and most accessible source of information and referral for help.

## 3. Aims of the study

This study had two aims. Firstly, the study aimed to assess the effect of a structured, time-limited, curriculum-based, peer-led educational programme on first-year high school learners compared to learners who have not received such an intervention in the Western Cape, South Africa. Secondly, the study sought to determine the feasibility of supporting peer-education programmes to reliably deliver risk reduction interventions in school settings in the Western Cape.

## 4. Methods

### 4.1. Implementation of the intervention

The peer-education programme was implemented by the Western Cape Department of Health and the Western Cape Department of Basic Education in collaboration with nine non-government organisations (NGOs) (the implementing organisations^1^). Technical assistance and monitoring was provided by the Centre for the Support of Peer Education and the intervention was developed by the Centre as part of the Western Cape’s proposal to the Global Fund. The intervention builds on the efforts of the first five years of the Global Fund’s support to the province. The peer-education curriculum is intended to follow the guidelines as set out in the National Life Orientation Curriculum Statement and each lesson is designed to fit into a school setting.

The curriculum (see Table 1) called ‘Listen Up’ comprises seven-structured sessions of 35 to 45 minutes each delivered co-curricular during Life Orientation lessons. The curriculum addresses issues such as supporting peers, sexual decision-making, healthy relationships, HIV and AIDS risk, alcohol misuse and early pregnancy. The programme targets adolescents in Grade 8 (aged between 13 and 15) living in challenging environments as adolescents in this age group are most susceptible to peer influences (Steinberg & Monahan, 2007).

**Table 1.** ‘Listen Up’ curriculum coverage.

Lesson number	Lesson title	Desired outcome
Lesson 1	Homies and helpers	Introduction to peer education
Lesson 2	Making smart decisions	Improved decision-making
Lesson 3	For the love of it: Relationships	Developing healthy relationships
Lesson 4	Safe my mate: Reducing risky behaviours	Reducing risky behaviours
Lesson 5	No go: Waiting for sex	Promoting abstinence
Lesson 6	Teenage pregnancy	Discouraging early pregnancy
Lesson 7	How much is too much: Talking about drinking	Alcohol awareness

Peer educators, those tasked with facilitating the peer-education sessions, were selected by the implementing organisation working with the respective schools and were selected using varied criteria. Learners either volunteered and/or were nominated by educators at participating schools to participate in the peer-education programmes. There were no rigid criteria defining who could be a peer educator and hence peer educators included individuals who are more academically proficient and learners who are less proficient academically but enjoy speaking with peers on various topics. Some peer educators were mainly interested in learning something new or belonging to a new group. A total of 162 peer educators were trained. Initially, the programme was designed to have senior learners in Grade 10 and Grade 11 trained to facilitate peer-education programmes with Grade 8 and Grade 9 learners. However, concern arose as the senior peer educators would lose academic contact time as a result of delivering material to younger peers. The Western Cape Department of Basic Education introduced this programme as a new delivery model using same-age peer educators to educate their peers. Peer educators were supported by adult facilitators. The facilitator was tasked with supporting and supervising the peer educator and was responsible for ensuring the successful delivery of the programme. Facilitators were drawn from either within the school or the implementing organisation working with the school.

### 4.2. Intervention evaluation

#### 4.2.1. Study design

In order to assess effects of the programme over time, evaluation of the programme was conducted in three waves. The evaluation used a mixed-method research design. Quantitative data were collected using a survey questionnaire and the data and results are reported in this paper. Data were collected from cross sections of the population at baseline. Data were then collected immediately post intervention (‘time 1’) and five to seven months post intervention (‘time 2’). The primary indicators for the study are based on 10 scales sourced from published literature. Table 2 lists the indicator scales for future orientation, sensation-seeking, self-efficacy in sexual relations, HIV transmission knowledge, HIV prevention knowledge, HIV attitudes, sexual attitudes, decision-making, healthy relationships and social support.

**Table 2.** Overview of the indicators used and sample questions.

Scales	Number of items	Type of question	Sample question	Cronbach’s alpha	Source
Sensation-Seeking	8	Agree/disagree (5-point Likert-type scale)	I would like to explore strange or new places. I prefer friends who do things on the spur of the moment.	0.65	Hoyle, Stephenson, Palmgreen, Lorch, and Donohew (2002)
Self-Efficacy in Sexual Relations	8	Not sure/kind of sure/totally sure (3-point scale)	How sure are you that you could use a condom correctly or explain to your partner how to use a condom correctly? Imagine that you and your partner have been in a relationship, but you have not had sex. Your partner really wants to have sex. Still, you don’t feel ready. How sure are you that you could keep from having sex until you feel ready?	0.87	Basen-Engquist et al. (1999)
Future Orientation	6	Agree/ disagree (3-point scale)	Do you agree or disagree: Dreams can be achieved? Do you agree or disagree: It’s okay for people my age to not come to school when they don’t feel like it?	0.53	HSRC All Stars Questionnaire
HIV Transmission Knowledge	6	Yes/No/Don’t know (3-point scale)	Is it possible to transmit HIV by drinking from the same cup? Is it possible to transmit HIV from a mother to her unborn baby?	0.70	WHO (1988)
HIV Prevention Knowledge	9	Yes/No/Don’t Know (3-point scale)	Can HIV infection be prevented by sticking to one partner, having only one sexual partner? Can HIV infection be prevented by taking antiretroviral drugs?	0.78	WHO (1988)
HIV Attitudes	12	Agree/ disagree (3-point scale)	Do you agree or disagree: AIDS is cured by having sex with a virgin? Do you agree or disagree: A person with HIV can look healthy?	0.72	WHO (1988)
Sexual Attitudes	5	Agree/ disagree (3-point scale)	Do you agree or disagree: Having sexual intercourse with someone besides a steady partner makes a boy or girl ‘cool’ or popular? Do you agree or disagree: If I do not have sex, I will lose some of my friends?	0.67	Center for AIDS Prevention Studies (1994)
Decision-Making	20	Never/ Always (5-point scale)	When I have a decision to make, I think about the problem before I take action. When I have a decision to make, I prioritise my choices before making a decision.	0.97	Mincemoyer and Perkins (2003)
Healthy Relationships	6	Agree/ Disagree (5-point scale)	Do you agree or disagree: If a boy buys a girl presents, she must have sex with him? Do you agree or disagree: If a girl suggests using a condom to her partner, it suggests she does not trust him?	0.93	Swartz et al. (2010) (Vhutshilo 2 Intervention)
Social Support	23	Agree/ disagree (5--point scale)	Do you agree or disagree: My family holds me in high esteem? Do you agree or disagree: My friends look out for me?	0.86	Procidano and Heller (1983)

#### 4.2.2. Sampling

The peer-education programme was rolled out in 236 schools in the Western Cape. It was assumed that in each school there would be approximately 120 Grade-8 learners. A total of 28,320 learners were therefore expected to participate in the peer-education programme. In order to detect a meaningful difference in outcome scores between intervention and control schools, a minimum of 2216 learners from intervention schools were to be sampled based on an error rate of 2% and a 95% confidence level.

Nine implementing organisations rolled out the peer-education programme in 10 sub-districts in the Western Cape. Taking into account the number of schools in each sub-district, a proportionate number of schools to be sampled per sub-district was determined. A lower Grade 8 learner estimate of 85 learners was used rather than the initial assumption of 120 learners to allow for absentees and to take account of attrition in class sizes over the three waves of the study. These assumptions required 27 intervention schools to be selected. Intervention schools in each sub-district were randomly selected and, within each school, classes were randomly selected for each wave of the study. However, these assumptions did not always fit the school situation and researchers had to accommodate these differences when administering survey questionnaires. To meet the requirement for a representative sample, in some schools more than 85 learners were included, while in others fewer than 85 learners participated. This means that in some schools three out of five classes were included while in others all classes were included resulting in fluctuating numbers of learners which differ from the targeted numbers. A control group of eight schools (one-third the size of the intervention group) stratified and proportionally sampled by sub-district was also randomly selected. Overall, however, the sample size was met. The samples therefore constitute a cross-section of the specific school grade communities over time. A further assumption was made that the large sample size would provide an overall indication of attitudinal and behavioural trends in these schools over time.

At baseline, 2904 learners were surveyed (2225 in the intervention group and 679 in the control group). Immediately post intervention (time 1), 2594 learners were surveyed (2036 in the intervention group and 558 in the control group). At five to seven months post intervention (time 2), 2211 learners were surveyed (all of whom were from intervention schools).

#### 4.2.3. Data collection

Data were collected through a single self-administered survey questionnaire offered in English, Afrikaans and Xhosa. Surveys were translated and back translated and piloted prior to initial use among the sample groups to rectify errors and address misunderstandings. Measured variables include participant demographics, community context; self-reported levels of sexual activity; exposure to peer education; delivery of the peer-education programme; sexual and HIV knowledge; attitudes and behaviour; decision-making; social support and positive social behaviour.

Trained fieldworkers facilitated the administration of the survey at the respective schools. Questionnaires were completed during the school day. The data collection was dependant on timeous access to the schools as well as the implementation of peer-education lessons (co-ordinated by the implementing organisations). Data collection began in February 2012 and ended in June 2013. The survey was administered at three time intervals: at baseline before the intervention in 2012, immediately post intervention in 2013 and delayed post-test (five to seven months later). A control group, who did not receive the peer-education programme, was measured at baseline and in the time frame of the immediate post-test intervention.

### 4.3. Ethical considerations

Permission to conduct the evaluation study was obtained from the Western Cape Education Department. Ethics approval was obtained from the Research Ethics Committee of the Human Sciences Research Council (HSRC), which is nationally accredited by the South African Government. Approval was sought for the use of the parent/guardian consent documents in the study as well as for the research instruments. Steps were taken to ensure confidentiality and anonymity for all research participants. Assent was obtained from participants under the age of 18 and informed consent obtained from their parents/guardians.

## 5. Data analysis

SPSS version 20 was used for data entry and analysis. Given the relevance of ‘sex’ for the research and for various analyses, participants who did not indicate their sex were excluded from the analysis.

### 5.1. Testing the reliability of indicator scales

Comparative analysis was conducted between control and intervention schools using an analysis of variance (ANOVA) for each of the 10 scales for the three waves of the study. Mean values of the control schools group were compared to those of the intervention schools group at baseline and post-test using ANOVA.

Cronbach’s alpha scale reliability test was conducted on each scale to measure their overall consistency. Items resulting in inconsistency of the scales were systematically removed to improve reliability. Scales with a Cronbach’s alpha value of 0.5 and above were accepted (see Table 2 for alpha values).

## 6. Results

The results are presented in two parts. The first part covers descriptive results comprising participant demographics, community context and exposure to and rating of peer education. The second part reports on the comparative analysis conducted between intervention and control schools. Given that the study was conducted on three cross-sectional samples of the school populations, descriptive results are provided for each sample group.

### 6.1. Demographic and socio-economic characteristics of participants

Sampled learners were aged between 10 years and 17 years or older. Although the intervention was initially targeted at adolescents aged from 10 to 13 years of age, it was decided that adolescents older than 13 in Grade 8 would be included in the study so as not to further disadvantage those who, for whatever reason, were not in the appropriate grade for their age. At baseline, the modal age of learners was 13 years old. For the immediate post-intervention and delayed post-intervention samples, the modal age was 14 years old. Table 3 provides a detailed sex and age breakdown for each sample group.

**Table 3.** Age and sex distributions.

	Time 0 Sample			Time 1 Sample			Time 2 Sample		
	*N* = 2904			*N* = 2594			*N* = 2211		
	Within age sex breakdown (%)		Age group proportion of total sample (%)	Within age sex breakdown (%)		Age group proportion of total (%) sample	Within age sex breakdown (%)		Age group proportion of total (%) sample
	Male	Female	Total	Male	Female	Total	Male	Female	Total
10 years	21	79	<1	77	23	1	50	50	<1
11 years	37	62	1	38	62	1	0	100	<1
12 years	37	63	10	27	73	4	55	45	1
13 years	37	63	35	39	61	26	36	63	10
14 years	43	57	23	44	56	36	40	60	36
15 years	52	48	12	49	50	16	47	53	27
16 years	59	41	4	59	41	6	53	47	12
+17 years	56	44	14	56	44	11	57	43	13
**Total**	**43**	**56**	**100**	**45**	**55**	**100**	**45**	**54**	**100**

At baseline, most learners identified themselves as black/African, followed by coloured. In the immediate post-intervention and delayed intervention samples, most learners identified themselves as being coloured. The number of white and Indian/Asian learners across samples was relatively small. Details pertaining to the sample can be found in Table 4. Samples selected from each school ranged in size from 15 to 161 learners across the three samples included in the study. Sex was almost equally represented in each of the school samples.

**Table 4.** Sample socio-economic characteristics.

Socio-economic characteristic	Time 0 Sample	Time 1 Sample	Time 2 Sample
	%	%	%
Population groups			
Black/African	50	45	43
White	3	3	1
Coloured	41	47	51
Indian/Asian	<1	<1	<1
Other	<1	1	1
No response	5	3	3
Lives with at least one parent	86	86	85
Main person who looks after you			
Biological mother	39	44	45
Biological father	5	6	5
Both parents	26	32	30
Household size (mean)	5.35	5.20	5.2
Dwelling type			
Formal house/flat (with an inside toilet)	78	81	82
Informal house	17	15	15
Traditional house	5	4	3
Household economic status			
Not enough money for food	16	9	9
Enough money for food but not enough money for other basic items such as clothes	18	15	14
Enough money for food and clothes but are short for other things	37	27	30
Enough money for food and clothes and also a bit extra for other things	29	50	47

Across all three samples, most learners reported living with at least one of their parents with the majority being cared for by their biological mother. The mean household size was just over five people across all three samples. Roughly 80% of learners in all sample groups lived in formal housing followed by informal settlements. Just over a third (37%) of learners in the baseline sample indicated that their families had enough money for food and clothes but are short for other things, while half of the immediate and delayed post-intervention samples indicated that their families had enough money for essentials and also had a bit extra for other expenses (50% and 47% respectively).

### 6.2. Community context

#### 6.2.1. Issues facing adolescents

Drug and alcohol abuse was highlighted as the most important issue facing adolescents in all three samples, followed by teenage pregnancy. HIV and AIDS were reported by roughly 14% across samples to be an important issue facing adolescents in their communities. Involvement in social activities encompassed a range of activities both around the house and in the community. In terms of activities engaged in on a daily basis, across all three samples, learners primarily watched television.

#### 6.2.2. Access to health services

Health services, in the form of a hospitals or clinics where sexual reproductive health services could be accessed, were more often accessed by female learners compared to male learners. This may be a result of clinics being perceived as more female ‘friendly’ than male ‘friendly’. It should also be noted that most learners did not make use of these services. Of those learners who have made use of these services, just over half were female across the three sample groups and four out of five indicated that they would use the service again across all sample groups.

### 6.3. Sources of HIV and AIDS knowledge

Across the samples, in the year preceding the study, most learners obtained information about HIV and AIDS from their teachers. This is followed by clinics as a source of information. Learners also indicated that they believed HIV and AIDS-related information obtained from teachers the most.

### 6.4. Exposure to peer education

Participants were asked about their exposure to peer-education programmes in the past. Prior to the intervention (at baseline), 18% of learners had engaged in peer-education programmes as participants with a 14% of these individuals being involved as peer educators.

Following the intervention, the majority of peer educators had engaged in both one-on-one meetings and classroom discussions with the peer-education facilitator (adult) once a week. A sizeable portion of peer educators had no interaction with their adult facilitators in the classroom or on a one-on-one basis across both post-intervention samples, although this decreased over time. Tables 5 and 6 display these findings.

**Table 5.** Percentage of learners engaging with their peer educators in various activities post intervention.

		Listening/discussing during peer-education lessons of the month	Group discussion	One-on-one meeting
Time 1 sample	Not involved	16	22	32
	Once a month	17	15	18
	Once a week	37	35	28
	3+ times a week	29	29	22
Time 2 sample	Not involved	17	25	37
	Once a month	20	15	15
	Once a week	39	37	28
	3+ times a week	24	24	20

**Table 6.** Frequency of peer educators’ engagement with their adult facilitators during peer-education programmes post intervention.

	Time 1 sample				Time 2 sample			
	*N* = 319 (Classroom discussions) *N* = 299 (One-on-one meetings)				*N* = 250 (Classroom discussions) *N* = 233 (One-on-one meetings)			
Portion (%) of engagement	Once a month	Once a week	3+ times a week	Never	Once a month	Once a week	3+ times a week	Never
Classroom discussion	18	39	31	12	16	40	33	10
One-on-one meeting	13	40	28	19	11	42	26	21

### 6.5. The quality and rating of peer-education programmes

In order to assess the quality of peer-education programmes, learners in intervention schools were asked a number of questions pertaining to the availability of adult facilitators and the content and coverage of the classes. While just over half of the sampled students in the intervention schools at time 2 and 3 felt the need to have a one-on-one discussion with their peer-education facilitator, over two-thirds were unable to do so. The most often reported reasons were that the adult facilitator could not be found, the facilitator had to see another class or the learner was afraid to approach the facilitator.

In terms of content coverage, most learners (93% of the intervention school respondents at time 1 and time 2) found that the classes on HIV and AIDS increased their knowledge and understanding of the topic. Most learners found the materials used in the programmes useful (as reported by 89% of the respondents at time 1 and 85% of respondents at time 2). The majority of learners also felt that it provided interesting information on HIV and AIDS (reported by 88% of the respondents at time 1 and 86% of the respondents at time 2) and it was easy to understand (reported by 85% of the respondents at time 1 and time 2). Nevertheless, 40% to 50% of the learners found some of the materials included in the programme ‘embarrassing’.

## 7. Comparative analysis

Comparative analyses using four ANOVAs were conducted to detect any changes and differences between the control and intervention groups as well as between the intervention groups across the samples. Differences were noted between the scores of the intervention and control groups at baseline (see the intervention and control group baseline comparison below in Table 7). The differences in scores indicate that the two groups cannot be considered comparatively at baseline. Hence, the outcomes between control and intervention groups immediately post intervention cannot be validated and were excluded from the analysis. A comparison had to be drawn between differences in scores of the intervention schools at baseline (time 0), immediately post intervention (time 1) and delayed post intervention (time 2) respectively. The first ANOVA considers the intervention and control groups at baseline. The remaining ANOVAs consider differences among the intervention school groups. These results are presented in Table 7.

**Table 7.** Results of ANOVA.

Comparison type	Comparison points	Statistically significant indicators					
		Sensation-seeking	Self-efficacy in sexual relations	Future orientation	HIV transmission knowledge	HIV prevention knowledge	Healthy relationships
Intervention -control group	Time 0 comparison	4.353*	4.049*				
Intervention group	Time 0 – time 1 comparison		9.173*	3.840*	16.691**	6.423**	6.6261*
	Time 1 – time 2 comparison		4.05*				
	Time 0 – time 2 comparison		26.31*		35.11*		

*p < .05.

**p < .01.

***p < .001

### 7.1. Intervention and control group: time 0

When analysing the intervention and control school participants at time 0, the results of the ANOVA show two significant results for sensation-seeking and self-efficacy in sexual relations. For both scales, significantly higher means were noted for control schools when compared to the intervention schools. Since the schools were selected at random, it was expected that there would be no significant variation between the two groups of schools with regard to the measured indicators.

### 7.2. Intervention groups: time 0 – time 1 comparison

Results of the ANOVA for the intervention schools at time 0 (baseline) and time 1 (immediately post intervention) indicate significantly higher means for five indicators immediately post intervention compared to the baseline for the intervention schools. These indicators include future orientation, self-efficacy in sexual relations, knowledge regarding HIV transmission, knowledge regarding HIV prevention and knowledge in terms of a healthy relationship. This, therefore, may imply that the peer-education intervention positively changed knowledge, attitudes, beliefs and behaviour with regard to these indicators in the short term.

### 7.3. Intervention group: time 1 – time 2 comparison

Results for the intervention group at time 2 sample (delayed post intervention) indicated a significantly higher mean for self-efficacy in sexual relations when compared to values recorded for the time 1 sample (immediate post-intervention). This suggests that longer standing improvements in self-efficacy in sexual relations occurred following the intervention.

### 7.4. Intervention group: time 0 – time 2 comparison

The ANOVA conducted for the intervention group at time 0 (baseline) and time 2 (delayed post intervention) shows a significantly higher mean for self-efficacy and HIV transmission knowledge. This indicates more positive self-beliefs and improved HIV transmission knowledge post intervention.

A summary of the main findings of the study are presented in Table 7.

In the short term, statistically significant improvements are noted for 5 of the 10 indicators. In the longer term, the peer-education programme appears to be effective in increasing learners’ knowledge with respect to the various means by which HIV is transmitted in addition to improving their self-efficacy in sexual relations. This suggests that the learners’ awareness of their ability to protect themselves from HIV risk, through a combination of abstinence, delaying sexual debut and using prophylactics, has increased.

## 8. Concluding discussion

This study aimed to evaluate a peer-education programme aimed at improving the knowledge, attitudes, perception and behaviours related to risky behaviours for adolescents entering high school. Results considered grade-wide changes in terms of 10 indicators. Only a small portion of the sampled learners felt that HIV and AIDS is a key challenge faced in their community. This may mean that the sampled adolescents are not aware of the implications of HIV and AIDS for young people in the country. It appears that the other challenges of drug and alcohol abuse and teenage pregnancy specifically, which were most often reported by the learners, manifest themselves physically much sooner and more severely and are therefore more apparent to the adolescents.

Learners’ knowledge of HIV transmission methods and self-efficacy in sexual relations increased in the long term. While it may be possible that other interventions and/or community effects contributed to this increase, the sample covered 27 schools across a number of sub-districts in the Western Cape and it is unlikely that all individuals would have been affected by other influences.

Significant results noted for sensation-seeking and self-efficacy in sexual relations at baseline suggest that there may be differences in the control and intervention schools that were not apparent during the random selection process and this may have an impact on this evaluation. Any differences between these two categories of schools must take into account these pre-existing differences.

Improving adolescents’ self-efficacy in sexual relations, and the associated sense of personal control, can be seen as a protective factor and may be an important means of empowering adolescents to make more informed sexual decisions. The significant long-term findings on self-efficacy may be related to the scope of the peer-education programme delivered. The programme targeted a broad range of topics attempting to improve not only knowledge related to HIV and AIDS but also attitudes and behaviours of adolescents in risky environments. It may be that many other school-based programmes focus on knowledge and targeted behaviours such as drug and alcohol use. These programmes may not focus on developing self-confidence and personal growth. By including the topic of developing self-efficacy in sexual relations, which may have been poorly addressed in the past, this peer-education programme exposed learners to a new area related to risky behaviours. This has evidently led to the significant long-term improvements noted. A similar finding was made by Mukoma et al. (2009).

While the programme was able to show significant improvements on half of the indicators immediately following the implementation of the programme, the methods used did not ensure long-standing improvements. This problem has also been noted by a number of meta-studies (Chandra-Mouli, Lane, & Wong, 2015). The material or methods employed in this programme may have been effective in creating awareness in the short term but less effective in improving knowledge and changing behaviours in the long term. Additionally, response rates for sensitive questions, such as sexual experience, were low and inconsistent. A review of research on sensitive questions in surveys found that misreporting sensitive information is common and the magnitude of misreporting is related to individual factors as well as survey design (Tourangeau & Yan, 2007). This may indicate that the use of a survey tool to bridge gaps on sensitive topics may have not been as successful as intended.

For peer educators to successfully deliver the programme, an enabling environment needs to be created with essential support provided by peer-education facilitators. In this programme, 10–20% of peer educators did not meet regularly with their facilitators, suggesting that the support structures put in place for peer educators were not comprehensive and may have affected the outcomes of the programme. Given these challenges, as well as the finding that learners trust information about HIV and AIDS received from clinics and teachers the most, programmes should consider involving teachers in peer-education programmes. Visser (2007) makes a similar observation.

The role of the individual in the success of a peer-education programme should not be overlooked. The personal skills and abilities of the peer educator in delivering each class are crucial. Since statistically significant differences were only noted for 2 of the 10 indicators in the long run, it may also be that the programme placed too many responsibilities on the peer educator or that the use of same-age peer educators is not an effective delivery model. The success of a peer­-education programme ultimately lies in the ability to implement such programmes. Support for such a programme is required at various levels, namely the implementing organisations, peer-educator facilitators and peer educators, to be an effective tool for improving learners’ outlook on risky behaviours.

### 8.1. Limitations of the study

In the course of the analysis, several limitations became apparent. Firstly, the cross-sectional design of the study placed limitations on the interpretation of results and conclusions drawn. This design did not allow individuals and related outcome measures to be assessed over time. Secondly, although school samples were drawn randomly, differences in the baseline samples between the intervention and control groups indicate the presence of pre-existing differences that were not considered. This led to limitations in terms of the conclusions that can be drawn from this study. Thirdly, learners indicated that the survey was too long and this was seen in the inconsistent completion of some questions by learners. These issues affect the internal validity of the study and limit the conclusions that can be drawn about the effects of this programme on changing attitudes and behaviours.

## 9. Recommendations

A number of recommendations stemmed from the analysis of the peer-education programme rolled out in the Western Cape. Conceptually, simpler, more attainable goals need to be set for programmes targeted at adolescents. As a period of transition, adolescence is a stressful and complex phase. Peer educators in this age group therefore require more support from adult peer-education facilitators to ensure processes are correctly followed and the desired outcomes of the intervention are achieved. As relevant literature and recent evaluations indicate, there should be at least a two-year age gap between peer educators and peer learners. Peer educators struggle with issues of trust with peers of the same age. The use of youth development NGOs to implement and support peer-education programmes in school appears effective and should be continued in future programmes. Given the challenges faced by learners in meeting with their adult facilitators, the learning programme should be enhanced with a focus on facilitation, referral and advocacy skills.

## Note

1. Through an open tender process, the Western Cape Department of Education and the Western Cape Department of Health appointed a number of NGOs to provide individuals who are trained to implement peer education.
